# Molecular Insight into the Anti-Inflammatory Effects of the Curcumin Ester Prodrug Curcumin Diglutaric Acid In Vitro and In Vivo

**DOI:** 10.3390/ijms21165700

**Published:** 2020-08-09

**Authors:** Rianthong Phumsuay, Chawanphat Muangnoi, Peththa Wadu Dasuni Wasana, Hasriadi, Opa Vajragupta, Pornchai Rojsitthisak, Pasarapa Towiwat

**Affiliations:** 1 Inter-Department Program of Pharmacology, Graduate School, Chulalongkorn University, Bangkok 10330, Thailand; Rianthong.P@student.chula.ac.th; 2 Institute of Nutrition, Mahidol University, Salaya, Nakhon Pathom 73170, Thailand; chawanphat.mua@mahidol.ac.th; 3 Pharmaceutical Sciences and Technology Program, Faculty of Pharmaceutical Sciences, Chulalongkorn University, Bangkok 10330, Thailand; dasuniwasana@ahs.ruh.ac.lk (P.W.D.W.); adhiehasri@gmail.com (H.); 4 Research Affairs, Faculty of Pharmaceutical Sciences, Chulalongkorn University, Bangkok 10330, Thailand; opa.vaj@mahidol.ac.th; 5 Department of Food and Pharmaceutical Chemistry, Faculty of Pharmaceutical Sciences, Chulalongkorn University, Bangkok 10330, Thailand; pornchai.r@chula.ac.th; 6 Natural Products for Ageing and Chronic Diseases Research Unit, Chulalongkorn University, Bangkok 10330, Thailand; 7 Department of Pharmacology and Physiology, Faculty of Pharmaceutical Sciences, Chulalongkorn University, Bangkok 10330, Thailand

**Keywords:** curcumin, curcumin diglutaric acid, inflammation, carrageenan-induced paw edema

## Abstract

Curcumin diglutaric acid (CurDG), an ester prodrug of curcumin, has the potential to be developed as an anti-inflammatory agent due to its improved solubility and stability. In this study, the anti-inflammatory effects of CurDG were evaluated. The effects of CurDG on inflammatory mediators were evaluated in LPS-stimulated RAW 264.7 macrophage cells. CurDG reduced the increased levels of NO, IL-6, and TNF- α, as well as iNOS and COX-2 expression in cells to a greater extent than those of curcumin, along with the potent inhibition of MAPK (ERK1/2, JNK, and p38) activity. The anti-inflammatory effects were assessed in vivo by employing a carrageenan-induced mouse paw edema model. Oral administration of CurDG demonstrated significant anti-inflammatory effects in a dose-dependent manner in mice. The effects were significantly higher compared to those of curcumin at the corresponding doses (*p* < 0.05). Moreover, 25 mg/kg curcumin did not exert a significant anti-inflammatory effect for the overall time course as indicated by the area under the curve data, while the equimolar dose of CurDG produced significant anti-inflammatory effects comparable with 50, 100, and 200 mg/kg curcumin (*p* < 0.05). Similarly, CurDG significantly reduced the proinflammatory cytokine expression in paw edema tissues compared to curcumin (*p* < 0.05). These results provide the first experimental evidence for CurDG as a promising anti-inflammatory agent.

## 1. Introduction

Macrophage activation plays a primary role in host immune defense mechanisms, specifically in the inflammatory response. Inflammation is a key response of the body that defends the body’s tissues against endogenous and exogenous stimuli, such as tissue damage, pathogens, and chemical irritants. The cardinal signs of inflammation include redness, heat, swelling, and pain, which lead to compromised functionality of tissues and organs [1]. The inflammation process is associated with the activation of nuclear factor kappa B (NF–κB) and phosphorylation of mitogen-activated protein kinases (MAPKs), namely p-38, c-Jun N-terminal kinases (JNKs), and extracellular-signal-regulated kinases (ERK1/2), resulting in the production and release of proinflammatory cytokines, including interleukin-1 beta (IL-1β), interleukin-6 (IL-6), and tumor necrosis factor alpha (TNF-α); and increased levels of inflammatory mediators, namely nitric oxide (NO) and prostaglandin E2 (PGE2), which are generated by inducible nitric oxide synthase (iNOS) and cyclooxygenase-2 (COX-2), respectively [2]. Even though inflammation is considered as a physiological response to defend the host against both external and internal stimuli, it also contributes to the pathophysiology of various chronic diseases, including rheumatoid arthritis, autoimmune disorders, atherosclerosis, and cancer [3] when these protective responses dysregulate. Therefore, the downregulation of the inflammatory response would be a surrogate approach for the treatment and prevention of pathological complications associated with chronic inflammatory diseases. Even though non-steroidal anti-inflammatory drugs (NSAIDs) are used in the management of acute inflammation, their undesirable effects have led to the search for new compounds from plants to be used in the prevention and treatment of inflammatory disorders with improved safety profiles.

**Figure 1:**
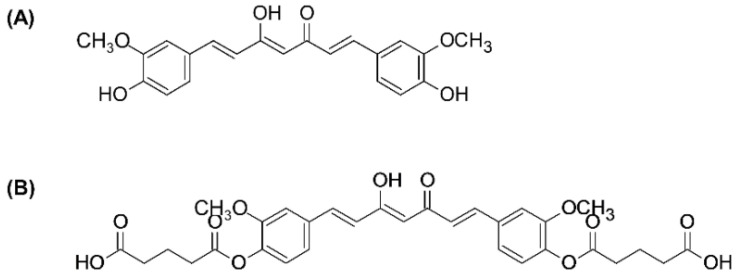
Chemical structures of (**A**) curcumin and (**B**) curcumin diglutaric acid (CurDG).

Curcumin (Figure 1A) is a major bioactive phytochemical contained in the rhizome of turmeric (*Curcuma longa* L.), which has several pharmacological activities, including antioxidant, anti-inflammatory, anticancer, and anti-infective properties [4]. Based on previous research, in vitro studies that indicate curcumin inhibits the production of NO, TNF-α, and IL-1β and suppresses NF-κB activation, which regulates the expression of genes in the inflammatory process [5]. In addition, curcumin downregulated the activity of COX-2 and iNOS enzymes related to inflammation. Many studies have investigated the anti-inflammatory effects of curcumin in animal models. One study reported that oral administration of curcumin inhibited edema induced by carrageenan at doses of 50–200 mg/kg in mice [6]. Moreover, curcumin has demonstrated beneficial effects in various diseases associated with inflammation, such as diabetes mellitus [7], myocardial infarction [8], and migraine [9]. While curcumin has many advantageous pharmacological properties, its development as a therapeutic agent is limited on account of its low aqueous solubility, poor absorption, and metabolic instability, which lead to poor oral bioavailability.

The prodrug approach has been applied to overcome the poor physicochemical and biopharmaceutical properties of numerous phytochemicals. Previously, we synthesized a glutaric ester prodrug of curcumin, the so-called curcumin diglutaric acid (CurDG) (Figure 1B), to improve the solubility of curcumin. We found that CurDG and curcumin were soluble in water at about 7.48 and 0.07 μg/mL, respectively, indicating about 100 times higher water solubility of CurDG compared to that of curcumin. In biological buffers at body temperature, curcumin and CurDG are sparingly soluble (<0.025 μg/mL), both in 0.1 M HCl and acetate buffer pH 4.5. The solubility of CurDG in phosphate buffer (pH 6.8) is greater than that of curcumin, at 1.43 μg/mL and 0.025 μg/mL, respectively. For the stability study, the hydrolysis rate (K*_obs_*) of CurDG at physiological pH 7.4 was found to be 2.62 h^−1^, which is higher the pH values of 1.2 and pH 4.5 (K*_obs_* 0.048 and 0.033, respectively). This indicates the possibility of CurDG to extend the degradation time of the released curcumin, facilitating the gradual absorption of CurDG through the cell membrane before the fast release of curcumin in plasma. The K*_obs_* and half-life for CurDG hydrolysis in human plasma were 5.83 h^−1^ and 0.12 h, respectively, while the CurDG prodrug was totally converted to curcumin in human plasma within 2 h [10]. Therefore, CurDG may have the potential to be developed for clinical applications, especially in the development of anti-inflammatory agents. However, the anti-inflammatory effect of CurDG has not been well reported. Hence, the aim of this study is to investigate the effects of CurDG on the proinflammatory cytokines in lipopolysaccharide (LPS)-stimulated RAW 264.7 cells, along with its anti-inflammatory activity in a carrageenan-induced mouse paw edema model, to provide evidence that this prodrug has better anti-inflammatory potential than curcumin.

## 2. Results

### 2.1. Cytotoxic Effects on Cell Viability of RAW 264.7 Cells

**Figure 2:**
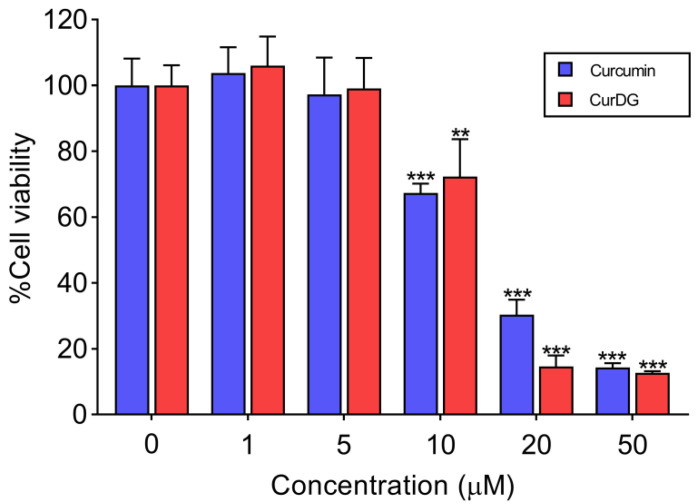
Effects of curcumin and CurDG on the cell viability of RAW 264.7 macrophage cells. Cells were treated with curcumin or CurDG at 1–50 μM and incubated for 24 h. Data show the mean ± SD values of three independent experiments. Note: ** *p* < 0.01 and *** *p* < 0.001 compared with the control group.

The cytotoxic effects of curcumin and CurDG on the viability of RAW 264.7 were evaluated using MTT assay. The results are presented as a percentage of cell viability. The results demonstrated that concentrations of up to 5 µM for both compounds had no cytotoxic effect on cell viability in RAW 264.7 cells (Figure 2). Therefore, we used the highest concentrations of curcumin and CurDG at 5 µM for subsequent experiments to ensure effective and sustained compound activity over the treatment period.

### 2.2. Effects on NO Levels in LPS-Stimulated RAW 264.7 Cells

**Figure 3:**
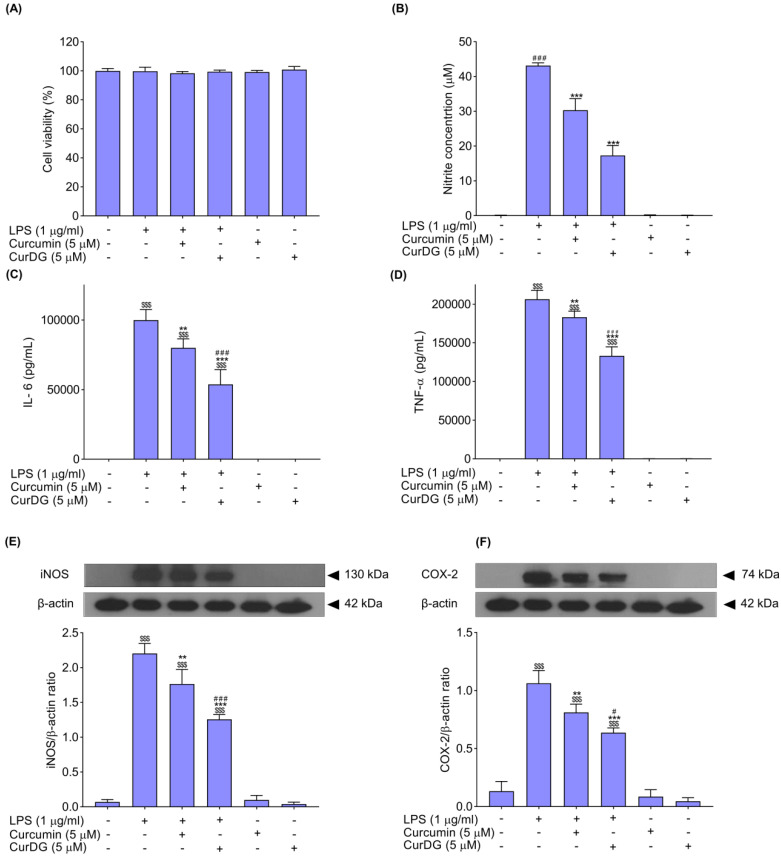
Effects of curcumin and CurDG on cell viability (**A**), NO production (**B**), and proinflammatory cytokine and mediator expression in LPS-stimulated RAW 264.7 cells. Cells were pre-treated with 5 μM curcumin or CurDG for 1 h, followed by the addition of 1 µg/mL LPS for 24 h. The culture medium was collected for NO determination and the cells were determined for cell viability with MTT assays. The IL-6 (**C**) and TNF-α (**D**) levels measured by ELISA assay are presented in pg/mL. The expression of iNOS (**E**) and COX-2 (**F**) proteins were determined by Western blot analysis, and these graphs were analyzed using the ImageJ software. The values of iNOS and COX-2 proteins were normalized with β-actin. The data present the mean ± SD, *n* = 3 of three independent experiments. Note: ^$$$^ *p* < 0.001 compared to the control group and ** *p* < 0.01; *** *p* < 0.001 compared to the LPS control group and ^#^ *p* < 0.05; ^###^ *p* < 0.001 significant difference between curcumin- and CurDG-treated groups.

To investigate the effects of curcumin and CurDG on the inhibition of LPS-stimulated NO production in RAW 264.7 cells, the quantifier of NO production was determined by comparing its absorbance with the standard curve of nitrite (NO_2_^−^). The results showed that the LPS-treated RAW 264.7 cells significantly increased the nitrite levels, peaking at 43.14 ± 0.81 μM, compared with the control group (0.07 ± 0.04). Pre-treatment of cells with both curcumin and CurDG at 5 μM for 1 h significantly decreased the levels of nitrite by 30% (30.33 ± 3.34 μM) and 60% (17.29 ± 2.90 μM), respectively, when compared to the LPS control group (Figure 3B). Additionally, treatment with only curcumin and CurDG did not affect the production of NO in RAW 264.7 cells (Figure 3B). The cytotoxic effects of curcumin and CurDG co-treated with LPS were measured using the MTT assay. The results showed that treatment with curcumin or CurDG with LPS had no cytotoxicity on RAW 264.7 cells, suggesting that the decrease in NO production from LPS-stimulated RAW 264.7 cells was not caused by the decrease in the cell population (Figure 3A). These results indicated that the CurDG had an anti-inflammatory effect on LPS-stimulated RAW 264.7 cells by decreasing NO production in LPS-stimulated RAW 264.7 cells at significantly higher levels than that of curcumin.

### 2.3. Effect on IL-6 and TNF-α Levels in LPS-Stimulated RAW 264.7 Cells

The effects of curcumin and CurDG on the levels of the proinflammatory cytokines (IL-6 and TNF-α) in LPS-stimulated RAW 264.7 cells were measured using ELISA. The result demonstrated that cells treated with LPS showed significantly increased production of IL-6 and TNF-α of up to 99,833 ± 7610 and 206,332 ± 11,623 pg/mL, respectively, compared to the control group (75 ± 1.8 and 83 ± 75, pg/mL respectively) (Figure 3C,D). Pre-treatment of cells with curcumin at 5 μM for 1 h significantly decreased the levels of IL-6 and TNF-α by 20% (79,917 ± 6487 pg/mL) and 11% (18,3016 ± 7922 pg/mL), respectively, when compared to the LPS-stimulated group (Figure 3C,D); while pre-treatment of cells with CurDG at 5 μM for 1 h significantly attenuated the increased levels of IL-6 and TNF-α by 46% (53,708 ± 10,567 pg/mL) and 35% (133,207 ± 11,653 pg/mL), respectively, when compared to the LPS-stimulated group. Additionally, treatment with only curcumin and CurDG did not affect the production of IL-6 and TNF-α in RAW 264.7 cells (Figure 3C,D). These results indicate that CurDG decreases the IL-6 and TNF-α secretion from LPS-stimulated RAW 264.7 cells to a greater extent than curcumin.

### 2.4. Effects on iNOS and COX-2 Expression in LPS-Stimulated RAW 264.7 Cells

To determine the effects of curcumin and CurDG on expression levels of iNOS and COX-2 in LPS-stimulated RAW 264.7 cells, these protein expressions were detected using Western immunoblotting. As shown in Figure 3E,F, protein expression of iNOS and COX-2 was significantly increased by approximately 32- and 8-fold, respectively, in RAW 264.7 cells stimulated with LPS. Pre-treatment of cells with curcumin at 5 μM for 1 h significantly decreased the expression of iNOS and COX-2 by 20% and 24%, respectively, when compared to the LPS-treated group (Figure 3E,F), whereas pre-treatment of cells with CurDG at 5 μM for 1 h significantly decreased the expression of iNOS and COX-2 by 43% and 40%, respectively, when compared to the LPS-induced group (Figure 3E,F). In addition, treatment with only curcumin and CurDG did not affect the expression of iNOS and COX-2 in control macrophage cells (Figure 3E,F). These results indicate that CurDG decreases the expression of iNOS and COX-2 from LPS-stimulated RAW 264.7 cells to a greater extent than curcumin.

### 2.5. Effectz on iNOS and COX-2 Expression in LPS-Stimulated RAW 264.7 Cells

**Figure 4:**
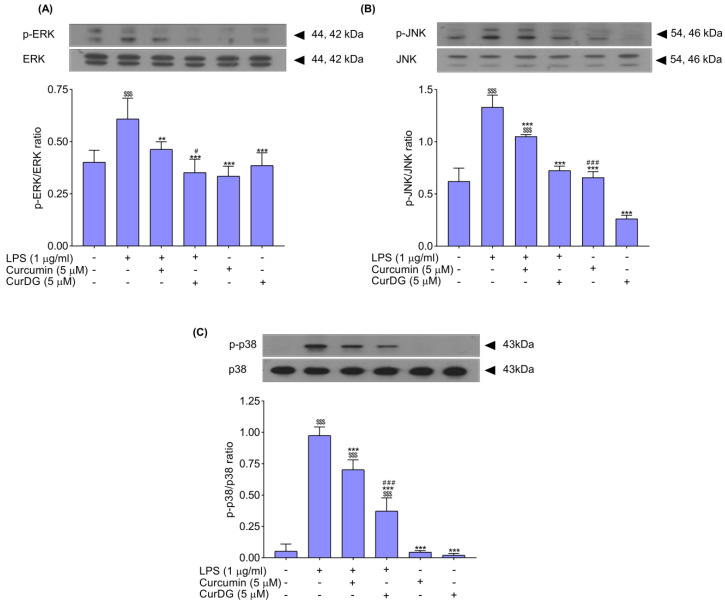
Effects of curcumin and CurDG on the expression of p-ERK (**A**), p-JNK (**B**), and p-p38 (**C**) in LPS-stimulated RAW 264.7 cells. Cells were co-treated with curcumin and LPS or CurDG and LPS for 24 h. The protein levels of phosphorylated and total forms of MAPKs were determined by Western blot analysis. The graphs were analyzed using the ImageJ software, and data show the mean ± SD of three independent tests. Phosphorylation levels of ERK1/2, JNK, and p38 were normalized with total levels of ERK1/2, JNK, and p38 densitometric values, respectively. Note: ^$$$^ *p* < 0.001 compared to the control group; ** *p* < 0.01 and *** *p* < 0.001 compared to the LPS-stimulated group; ^#^ *p* < 0.05 and ^###^ *p* < 0.001, significant differences between curcumin- and CurDG-treated groups.

The effects of curcumin and CurDG on intracellular signaling pathway protein MAPK expression, including ERK1/2, JNK, and p38, in LPS-stimulated RAW 264.7 cells were investigated using Western blot analysis. The LPS treatment significantly increased the expression of p-ERK, p-JNK, and p-p38 by approximately 1.5-, 2.1-, and 18.9-fold, respectively, in comparison with the control group (Figure 4A–C). Pre-treatment of cells with curcumin at 5 μM for 1 h significantly decreased the expression of p-ERK, p-JNK, and p-p38 by 24%, 21%. and 28%, respectively, when compared to the LPS-stimulated group (Figure 4); while pre-treatment of cells with CurDG at 5 μM for 1 h significantly decreased the expression of p-ERK, p-JNK, and p-p38 by 42%, 46%, and 62%, respectively, when compared to the LPS-stimulated group (Figure 4). Additionally, the treatment with only curcumin and CurDG did not affect the expression of p-ERK, p-JNK, or p-p38 in control cells (Figure 4). The results indicate that CurDG decreases the expression of p-ERK, p-JNK, and p-p38 from LPS-stimulated RAW 264.7 cells to a greater extent than curcumin.

### 2.6. Inhibitory Effects of CurDG on Carrageenan-Induced Mouse Hind Paw Edema

**Figure 5:**
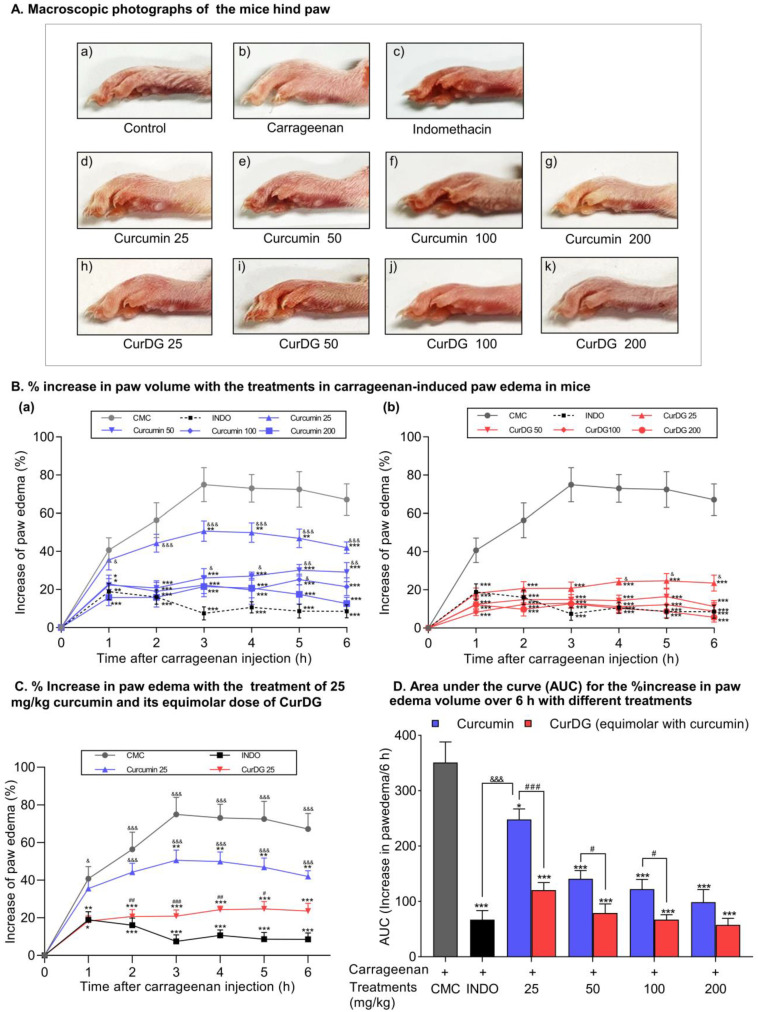
Anti-inflammatory effects of curcumin and CurDG on carrageenan-induced hind paw edema. (**A**) Morphological features of the left hind paw of each mice 6 h after carrageenan administration. (**B**) The percentage increase of paw edema after carrageenan injection following oral administration of 0.5% CMC, indomethacin (10 mg/kg), and curcumin (**a**) and CurDG (**b**) at doses of 25, 50, 100, and 200 mg/kg). (**C**) Effect of CurDG 25 on the % increase of paw edema compared to its respective controls. (**D**) The area under the paw edema effect–time curve (AUC 0–6 h). The data are the mean ± SEM for 8 mice. The results were analyzed using an ANOVA and Fisher’s LSD post hoc test. Note: * *p* < 0.05, ** *p* < 0.01, and *** *p* < 0.001 compared to the vehicle-treated control; ^#^ *p* < 0.05, ^##^ *p* < 0.01 and ^###^ *p* < 0.001, significant differences between curcumin- and CurDG-treated groups; ^&^ *p* < 0.05, ^&&^ *p* < 0.01, and ^&&&^ *p* < 0.001, statistical differences in the treatment groups compared with indomethacin-treated group (INDO).

The anti-inflammatory activities of curcumin and CurDG on the paw edema induced by carrageenan are shown in Figure 5. The morphological characteristics of mice paws observed at 6 h after carrageenan injection are shown in Figure 5A. Mice who received carrageenan in their left hind paws showed progressive increases in the volume of edema compared to the paw volume before injection (Figure 5A(a,b)). The morphologies of indomethacin-, curcumin-, and CurDG-treated paws showed reduced swelling, while swelling in the curcumin group remained higher compared to that of indomethacin- and CurDG-treated groups. As shown in Figure 5B, the subcutaneous administration of carrageenan produced a time-dependent increase in paw edema, which reached a maximum at 3 h (74.9 ± 9.0% increase in paw volume) and was maintained thereafter for 6 h, indicating acute inflammation. Oral administration of curcumin at doses of 25, 50, 100, and 200 mg/kg and equimolar doses of CurDG significantly reduced the development of the carrageenan-induced paw edema compared to that of the vehicle-treated group (*p* < 0.05) at 1–6 h (Figure 5B). The orally administered 25 mg/kg curcumin significantly reduced paw edema compared to the vehicle-treated group after only 3 h of induction (Figure 5B,C), with a maximal 32.3 ± 5.6% inhibition of paw edema at 6 h. However, CurDG at a dose equimolar to 25 mg/kg curcumin produced a greater reduction in paw edema, even after 1 h of carrageenan induction—the maximum effect (69.8 ± 4.3% inhibition of paw edema) was observed at 6 h (Figure 5B,C). 

For a clear comparison between compounds, the analysis of the edematous effect was plotted along a time curve and converted into the area under the curve (AUC) (Figure 5C). The anti-inflammatory effects of both curcumin and CurDG increased in a dose-dependent manner, with the highest dose of curcumin and its equimolar dose of CurDG having equal efficacy (*p* > 0.05). In contrast, the anti-inflammatory efficacy of CurDG at doses equimolar to 25, 50, and 100 mg/kg curcumin was significantly higher compared to that of curcumin (*p* < 0.05). Furthermore, the anti-inflammatory efficacy of all doses of CurDG, as well as curcumin except 25 mg/kg, was comparable with that of the reference drug indomethacin (10 mg/kg, orally). More specifically, CurDG at the lowest dose tested produced a significant anti-inflammatory effect, whereas curcumin did not produce a significant anti-inflammatory effect at the same dose level (25 mg/kg). The anti-inflammatory effect of CurDG 25 was comparable with that of curcumin at the dose levels of 50, 100, and 200 mg/kg. These results show that CurDG more effectively reduced paw edema in a dose-dependent manner compared to that of curcumin in mice with carrageenan-induced inflammation.

### 2.7. CurDG-Mediated Inhibition of Proinflammatory Cytokine Expression in Mouse Paw Edema Model

**Figure 6:**
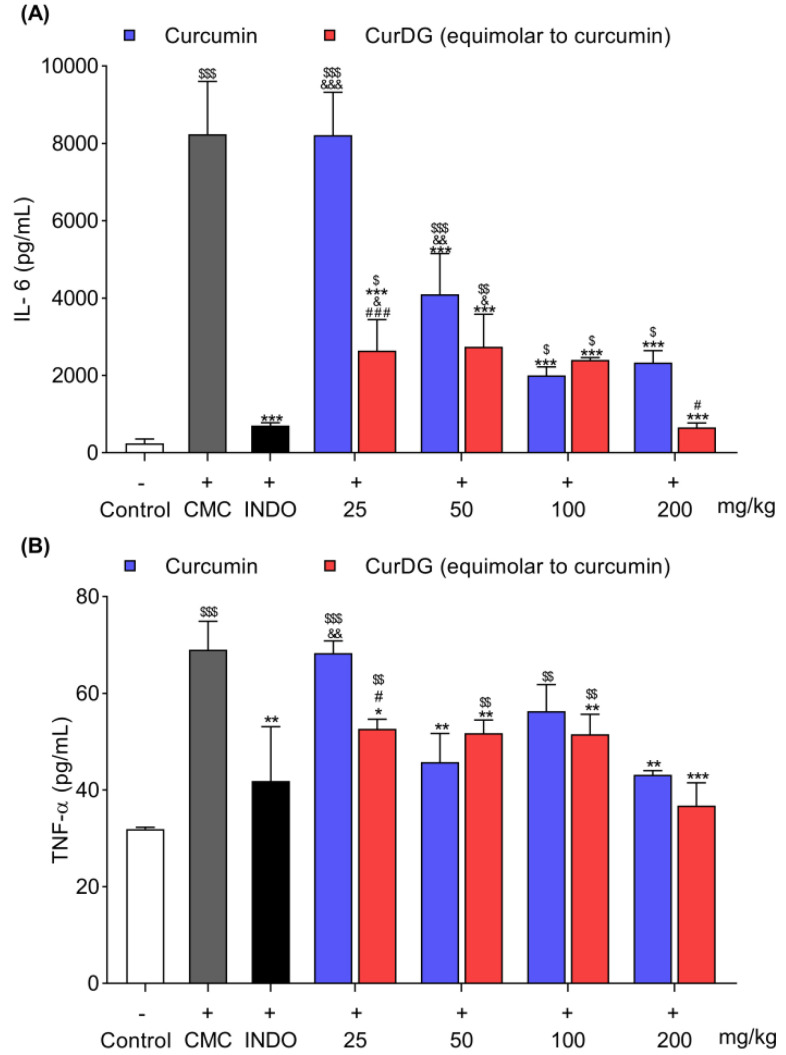
Effects of curcumin and CurDG on IL-6 (**A**) and TNF-α (**B**) levels in edema paw tissue. Results are shown as means ± SEM (n=8/group). Note: ^$^ *p* < 0.05, ^$$^ *p* < 0.01 and ^$$$^ *p* < 0.001 compared to the naïve group; * *p* < 0.05, ** *p* < 0.01, and *** *p* < 0.001 compared to the carrageenan group; ^#^ *p* < 0.05 and ^###^ *p* < 0.001 indicate significant differences between curcumin- and CurDG-treated groups; ^&^ *p* < 0.05 and ^&&^ *p* < 0.01 indicate statistical differences in the treatment groups compared with the indomethacin-treated group (INDO).

The molecular insight into the anti-inflammatory effects of CurDG was further investigated by analyzing the TNF-α and IL-6 levels—the proinflammatory cytokines of the inflamed paw. As shown in Figure 6, the injection of carrageenan significantly elevated the expression of both IL-6 (8239 ± 1361 pg/mL) and TNF-α (69.0 ± 5.9 pg/mL) (Figure 6A,B) in the inflamed paw compared to the control paw (243.7 ± 116.1 and 31.9 ± 0.4 pg/mL for IL-6 and TNF-α, respectively) (*p* < 0.001). The mice were administered orally with all four doses of CurDG equimolar to 25, 50, 100, and 200 mg/kg curcumin and showed significant reductions in carrageenan-induced proinflammatory cytokine release in paw tissues (*p* < 0.05) compared to the vehicle-treated group. The effect was comparable with indomethacin 10 mg/kg at the doses of 100 and 200 mg/kg for IL-6 expression and at all doses for TNF-α expression. Similarly, curcumin also significantly downregulated the expression of carrageenan-induced proinflammatory cytokines at doses of 50, 100, and 200 mg/kg. In agreement with the results of paw edema, curcumin 25 mg/kg did not significantly reduce the expression of either IL-6 (8211 ± 1111 pg/mL) or TNF-α (68.3 ± 2.5 pg/mL) in paw tissues compared to the vehicle control group. Interestingly, the inhibitory effect of CurDG at the dose equimolar to 25 mg/kg curcumin was significantly higher (2640 ± 804.6 and 52.6 ± 2.0 pg/mL, respectively, for IL-6 and TNF-α) than that of the parent compound, curcumin (*p* < 0.05). Above this, the inhibitory effects of CurDG at a dose equimolar to 25 mg/kg curcumin was comparable with those of curcmin at 50, 100, and 200 mg/kg dose levels. With respect to the reference drug, indomethacin, CurDG at all doses tested resulted in comparable (*p* > 0.05) reductions in TNF-α levels in paw tissues, except for the significantly lower inhibition of the IL-6 levels (*p* < 0.05) observed with CurDG doses equimolar to 25 and 50 mg/kg curcumin. Altogether, CurDG caused dominant inhibition in IL-6 levels compared to TNF-α levels in the carrageenan-induced paw tissues.

## 3. Discussion

The current study provides evidence of the improved anti-inflammatory effect of curcumin by its ester prodrug CurDG in both in vitro and in vivo models. CurDG in LPS-stimulated RAW 246.7 macrophage cells suppressed the levels of proinflammatory cytokines IL-6 and TNF-α; and the expression of proinflammatory mediators NO, COX-2, and iNOS via modulating MAPK pathways (ERK1/2, JNK, and p38) to a greater extent than curcumin. In line with the in vitro study, CurDG also exhibited higher anti-inflammatory effects in carrageenan-induced paw edema mice, where it significantly reduced the expression of proinflammatory cytokines (TNF-α and IL-6) in paw edema tissues compared to that of curcumin. 

A prodrug is an inactive form of the parental drug that undertakes biotransformation to an active form when administered to the body and is intended to increase the performance of the parent drug. This approach is utilized to improve the properties of the parent drug, such as its physicochemical and pharmacokinetic properties [11]. Recently, several prodrugs with excellent anti-inflammatory enhancement and better safety profiles compared to their parent drugs have been reported, including sulindac, parecoxib, nabumetone, and nepafenac, all of which are commercially available on the market [12]. 

Studies including clinical trials have demonstrated the effectiveness of natural products on decreasing proinflammatory responses in many diseases [13,14]. Owing to poor physicochemical and pharmacokinetic properties, there has been much attention paid to developing prodrugs from natural products with better bioavailability and stability profiles than their parent compound, such as resveratrol prodrugs [15], quercetin–amino acid conjugates (quercetin prodrugs) [16], scutellarin prodrugs [17], and curcumin prodrugs [10,18]. The curcumin prodrug CurDG has shown increased water solubility and an increased antinociceptive effect [10]. Here, the anti-inflammatory effect of CurDG was evaluated in vivo using carrageenan-induced edema and its underlying molecular mechanisms in both LPS-stimulated RAW 264.7 cells and inflamed mice paw tissues. Hence, this is the first study to compare the anti-inflammatory effects of CurDG with those of its parent compound, curcumin.

Inflammation is an essential response from the body that protects the tissues from both endogenous and exogenous stimuli. The activation of macrophages plays a crucial function in host immune defense mechanisms, specifically in inflammatory pathways. Macrophages, which are activated by pathogens and chemicals including LPS, bind with Toll-like receptors (TLR-4) present in macrophages, which in turn activate several downstream signaling pathways, including signal transduction pathway kinases [19], which eventually activate transcription factor NF–κB. This activation facilitates the translocation of NF–κB into the nucleus, where it binds with its response element, which then activates the expression of genes of several proinflammatory mediators [20]. During the state of chronic inflammation, these proinflammatory mediators are produced abundantly, initiating pathogenesis of the onset of numerous inflammatory disorders [21]. Moreover, LPS-stimulated macrophages lead to the phosphorylation of MAPK signaling proteins, including ERK1/2, JNK, and p38, which result in excessive secretion and expression of inflammatory mediators and proteins, including NO, TNF-α, and IL-6, as well as the expression of iNOS and COX-2. Together these mediators play an important role in the induction of inflammation [22]. The results of the present study also demonstrated that LPS exposure activated the secretion of NO, TNF-α, and IL-6, as well as the expression of iNOS and COX-2 in RAW 264.7 macrophage cells mediated via the phosphorylation of ERK1/2, JNK, and p38. Pre-treatment of cells with CurDG significantly inhibited all of these inflammatory cytokines and mediators compared to the parent drug, curcumin, indicating an improved anti-inflammatory effect of CurDG compared to curcumin. 

As CurDG was able to inhibit LPS-stimulated inflammatory reactions in RAW 264.7 macrophage cells as compared to curcumin, the effect was also investigated using an in vivo approach. The carrageenan-induced paw edema is a commonly used in vivo model for screening of anti-inflammatory agents [23]. Development of paw edema induced by carrageenan is described as a biphasic event; the early phase (2 h post carrageenan injection) is mediated by the secretion of inflammatory mediators (histamine and serotonin), which is correlated with enhanced vascular permeability. The second phase (3–4 h post carrageenan injection) principally involves neutrophil infiltration into the site of inflammation and the release of PGE2 and cytokines [24]. Following the injection of carrageenan, the anti-inflammatory reaction is normally evaluated for 6 h [25]. In agreement with previous studies [26], our study results demonstrated a polynomial increase in the edema size with the administration of carrageenan, where the size of edema reached a maximum after three hours of carrageenan injection. Furthermore, the anti-inflammatory effect of orally administered curcumin in carrageenan-induced paw edema has been evaluated previously, demonstrating its significant anti-inflammatory effects at doses of 25–400 mg/kg compared to the vehicle treatment [4,27]. Similarly, in the present study, oral administration of curcumin at 25, 50, 100, and 200 mg/kg effectively attenuated the carrageenan-induced paw edema in mice. Additionally, equimolar doses of CurDG produced significantly improved anti-inflammatory effects compared to curcumin at doses of 25, 50, and 100 mg/kg. Moreover, the antiedematous effect of CurDG at the dose equimolar to 25 mg/kg curcumin was comparable with that of the reference drug, indomethacin (10 mg/kg, p.o.), a well-recognized non-steroidal anti-inflammatory drug (NSAID)and cyclooxygenase (COX-1 and 2) inhibitor, as well as curcumin at 50, 100, and 200 mg/kg. Curcumin at the same dose showed significantly lesser efficacy compared to indomethacin, indicating the improved therapeutic efficacy of curcumin by formulating its prodrug CurDG. 

As per previous research findings, some of the synthesized curcumin–amino acid conjugate prodrugs also exhibited comparable anti-inflammatory activities to 10 mg/kg indomethacin (dose equimolar to 10.3 mg/kg curcumin) when administered to rats with carrageenan-induced paw edema [28]. However, in the previous study, the intraperitoneal route of administration was used. In line with that study, in our study the lowest dose of CurDG tested (dose mol equivalent to 25 mg/kg curcumin) showed comparable anti-inflammatory effects to indomethacin 10 mg/kg when administered orally. Moreover, CurDG 25 mg/kg showed more than 50% inhibition of paw edema at each time point tested. Hence, further studies are required using lower doses to gain a better understanding of the potency of CurDG relative to curcumin and to determine the dose that produces 50% inhibition of paw edema (ED_50_). 

The pathogenesis of carrageenan-induced inflammation is linked to the infiltration of immune cells, including macrophages, and their ability to release several proinflammatory cytokines, including TNF-α and IL-6 [23]. The transcription of these proinflammatory cytokines is mediated by the translocation of NF-κB into the nucleus via the activation of upstream pathways of MAPKs (ERK1/2, JNK, and p38). Additionally, this pathway also mediates the expression of various other mediators, including COX-2 and iNOS, which play crucial roles in the generation of acute inflammation [29]. In regards to the effect of carrageenan-induced paw edema on behavioral changes, pain hypersensitivity was induced after the intraplantar injection of carrageenan [30]. The findings of this study revealed the ability of the curcumin prodrug, CurDG, to reduce the cytokine levels in paw edema tissues compared to the parent drug alone. Hence, this effect could be attributed to the potent inhibition of the aforementioned intracellular signaling cascade, as shown in the in vitro study. Taken together, the results demonstrated the improved anti-inflammatory effect of curcumin against carrageenan-induced paw edema in mice using its ester prodrug CurDG.

## 4. Materials and Methods

### 4.1. Materials and Chemicals

Indomethacin, carrageenan, carboxymethylcellulose, lipopolysaccharide (LPS), 3-(4,5-dimethylthiazol-2-yl)-2,5-diphenyltetrazolium bromide (MTT), and other chemicals were purchased from Sigma Chemical (St. Louis, MO, USA). Curcumin and curcumin diglutaric acid (CurDG) were synthesized as previously described by Muangnoi et al. [10], by the Natural Products for Ageing and Chronic Diseases Research Unit, Faculty of Pharmaceutical Sciences, Chulalongkorn University, Bangkok, Thailand. Briefly, glutaric anhydride in dichloromethane (9 mmol) was added to a solution containing curcumin (4 mmol) and triethylamine (9 mmol) in dichloromethane at 40 °C with nitrogen. After refluxing for 2 h, 0.1 N HCl and dichloromethane were added to the reaction mixture. The organic layer was separated, washed with water, dried over anhydrous sodium sulfate, and evaporated until dry using a rotary evaporator. Finally, the residue was purified by crystallization from methanol to yield CurDG as a yellow solid. The chemical structure of CurDG was confirmed by 1H-NMR [10].

### 4.2. Cell Culture

The RAW 264.7 macrophage cell line, purchased from the American Type Culture Collection (ATCC, Rockville, MD, USA), was cultured in Dulbecco’s modified Eagle’s medium (DMEM) (Invitrogen, Grand Island, NY, USA) supplemented with 10% fetal bovine serum and 1% penicillin-streptomycin at 37 °C in a humidified incubator under 5% CO_2_.

### 4.3. Evaluation of Cytotoxic Effects of Curcumin and CurDG on Cell Viability of RAW 264.7 Cells

RAW 264.7 cells (1.5 × 10^4^ cells/well) were seeded in 24-well plates and incubated for 24 h. After incubation, the medium was removed and 1 mL of serum-free medium was added per well. Cells were then treated with 0.5% DMSO or various concentrations of curcumin (1, 5, 10, 20, and 50 µM) and its equimolar concentrations of CurDG. Cell viability was measured using the MTT assay by adding MTT solution (0.5 mg/mL in PBS), followed by incubation for 4 h. The absorbance was then measured at 540 nm. The results were presented as % cell viability by comparison with the control group.

### 4.4. Anti-Inflammatory Effects of Curcumin and CurDG on LPS-Stimulated RAW 264.7 Cells

RAW 264.7 cells were seeded in 6-well plates at a density of 1.5 × 10^6^ cells/well for 24 h. Then, cells were washed with phenol red-free medium and the medium was replaced with 2 mL of phenol red-free medium per well. The cells were then treated with 0.5% DMSO, curcumin, or CurDG at 5 µM (without toxicity) for 1 h, followed by stimulation with 1 µg/mL of LPS [31]. After incubation for 24 h, the culture medium was collected for determination of nitrite, TNF-α, and IL-6 levels. The cell pellets were collected for Western blot analysis 

#### 4.4.1. Nitrite Level Measurement (NO Assay)

The nitrite concentrations in the culture medium as an indicator of nitric oxide levels were analyzed by the Griess reaction by adding 100 μL of the culture medium to 50 μL of sulfanilamide (1% in 5% phosphoric acid) in each well of a 96-well plate, followed by incubation for 5 min in the dark. Then, 50 μL of *N*-1-Napthylenediamine dihydrochloride (NED) solution was added, incubated for 5 min, then the absorbance was measured at 520 nm. The concentration of NO was determined by comparison to the standard curve of NaNO_2_ [31].

#### 4.4.2. Determination of IL-6 and TNF-α Levels

The levels of IL-6 and TNF-α were determined with a mouse enzyme-linked immunosorbent assay (ELISA) kit, according to the manufacturer’s instructions (BioLegend, San Diego, CA, USA). IL-6 and TNF-α results for each sample were calculated from their respective standard curves.

#### 4.4.3. Western Blot Analysis

After treatment (Section 2.4), the cells were washed with ice-cold PBS and lysed by ice-cold RIPA lysis buffer containing a protease and phosphatase inhibitor cocktail (Roche, Mannheim, Germany). Then, centrifugation of the cell lysates at 12,000× *g* and at 4 °C for 10 min and the concentration of protein was determined by BCA assay. Equal amounts of each protein sample (20 μg) were separated using 10% sodium dodecyl sulfate–polyacrylamide gel electrophoresis, then the proteins were transferred onto a nitrocellulose membrane. Membranes were blocked with 5% skim milk for 2 h and incubated overnight with primary antibodies for iNOS, COX-2, phospho-P38, P38, phospho-ERK, ERK, phosphor-JNK, JNK, (1:1000), or β-actin (1:20,000) (Cell Signaling Technology, Danvers, MA, USA) at 4 °C. After 24 h, the membrane was incubated with antirabbit IgG conjugated to HRP secondary antibodies (1:2000) for 90 min. Specific protein bands were exposed to X-ray film and detected using enhanced chemiluminescence (ECL) (Bio-Rad, Hercules, CA, USA). The band densities were quantified using the Image J program and the intensity of the bands was expressed as a relative ratio of the specific proteins and β-actin or total forms of MAPKs.

### 4.5. Animals

Male ICR mice (18–25 g) were obtained from the Nomura Siam International, Thailand, and maintained at the animal facility of the Faculty of Pharmaceutical Sciences, Chulalongkorn University, Thailand. Animals were housed in a temperature-conditioned room (24 ± 2 °C and 40–60% humidity) with a 12/12 h light/dark cycle. All animals were provided with food and water ad libitum. All the experimental protocols were approved by the Institutional Animal Care and Use Committee of the Faculty of Pharmaceutical Sciences, Chulalongkorn University, Thailand (protocol number: 1833002, 12 March 2018), in agreement with the guidelines of use for laboratory animals for scientific purposes.

### 4.6. Drugs and Treatments

Mice were randomly divided into 10 groups (*n* = 8/group) as the vehicle control, indomethacin control, curcumin-treated group (4 doses), and CurDG-treated group (4 doses). Group 1 and 2 served as the vehicle control and indomethacin control, respectively. Groups 3, 4, 5, and 6 received 25, 50, 100, and 200 mg/kg of curcumin, respectively; while groups 7, 8, 9, and 10 received CurDG doses equimolar to 25, 50, 100, and 200 mg/kg curcumin, respectively. All drugs and treatments were freshly prepared and administered orally at a final volume of 10 mL/kg. CMC 0.5% in normal saline was used as the vehicle. Indomethacin was dissolved in CMC (0.5% in normal saline) and administered at 10 mg/kg. Both curcumin and CurDG were dissolved in 0.5% CMC. Curcumin at the doses of 25, 50, 100, and 200 mg/kg; and equimolar doses of CurDG 40.6, 81.1, 162.3, and 324 mg/kg, respectively, were used in this study. 

### 4.7. Carrageenan-Induced Paw Edema Test

The anti-inflammatory activity of the compounds was determined using the carrageenan-induced edema test, as described by Winter et al. [32]. Mice were administered with vehicle, indomethacin, or one of four different doses of curcumin or CurDG. After one hour, the mice were injected subcutaneously onto the plantar surface of the left hind paw with 1% λ-carrageenan in saline (50 µL/paw). The paw volume was determined at 0 h (volume of the paw before carrageenan injection, V_0_) and at 1, 2, 3, 4, 5, and 6 h after carrageenan injection (V_t_) using a plethysmometer (Model 7150, UGO Basile, Gemonio, VA, Italy). The paw edema was determined as the average increase in the paw volume compared to the control group, calculated according to the following formula [33]:

% increase of paw volume = ((V_t_ − V_o_)/V_o_) × 100 (1)

The anti-inflammatory effects of the compounds were determined as a percentage of inhibition of the paw edema in the treatment groups in comparison to the carrageenan control group [24]:

% inhibition of paw edema = ((V_t_ − V_o_)_control_ − (V_t_ − V_o_)_treatment_)/(V_t_ − V_o_)_control_ × 100 (2)

### 4.8. Determination of Proinflammatory Cytokines in Inflamed Paw Tissues 

Upon completion of the paw edema measurements, hind paws injected with carrageenan were collected. The hind paws were dissected at the level of the calcaneus bone and the soft tissue samples were removed and stored at –80 °C until use. The tissue samples were weighed and paw tissues (20% (*w*/*v*)) were homogenized in ice-cold PBS at pH 7.4. Then, the homogenates were centrifuged at 10,000 rpm for 10 min at 4 °C and the supernatants were collected. Three to five samples of supernatants were randomly picked to determine the concentrations of TNF-α and IL-6 and IL1-β using ELISA kits (BioLegend, San Diego, CA, USA), according to the manufacturer’ protocol.

### 4.9. Statistical Analysis

Results are presented as the mean ± SD values (*n* = 3) of three independent experiments for in vitro studies and as the mean ± SEM values (*n* = 8) for the in vivo studies. The statistical differences between groups were analyzed by one-way analysis of variance (ANOVA) followed by Fisher’s LSD post hoc test. Here, *p*-values < 0.05 were considered statistically significant. All statistical calculations were performed using Graph Pad Prism 7.0 (USA) statistical software.

## 5. Conclusions

In summary, the present study provides evidence that CurDG has a more potent anti-inflammatory effect than curcumin both in vitro and in vivo through suppression of inflammatory mediator production (IL-6, TNF-α, COX-2, and iNOS). The molecular mechanisms by which CurDG demonstrated its effects involve the modulation of the phosphorylation of MAPK signaling pathways, such as ERK1/2, JNK, and p38. The results provide the first experimental evidence for CurDG as a promising therapeutic agent for treating disorders characterized by inflammation. 

## Abbreviations

CurDG	Curcumin diglutaric acid
NF–κB	Nuclear factor kappa B
MAPKs	Mitogen-activated protein kinases
JNKs	c-Jun N-terminal kinases
ERK1/2	Extracellular signal-regulated kinases
IL-1β	Interleukin-1beta
IL-6	Interleukin-6
TNF-α	Tumor necrosis factor
NO	Nitric oxide
PGE2	Prostaglandine2
iNOS	Inducible nitric oxide synthase
COX-2	Cyclooxygenase-2
NSAIDs	Non-steroidal anti-inflammatory drugs
AUC	Area under the curve
TLR-4	Toll-like receptors
LPS	Lipopolysaccharide
MTT	3-(4,5-Dimethylthiazol-2-yl)-2,5-diphenyltetrazolium bromide
DMSO	Dimethyl sulfoxide
NED	*N*-1-Napthylenediamine dihydrochloride
ELISA	Enzyme-linked immunosorbent assay
ECL	Enhanced chemiluminescence
SEM	Standard error of the mean
CMC	Carboxymethyl cellulose
